# The Nasopharyngo‐Septal Butterfly Flap: A Novel Adjunct for Reconstructing Large Skull Base Defects

**DOI:** 10.1002/oto2.70016

**Published:** 2024-10-30

**Authors:** Moataz D. Abouammo, Maithrea S. Narayanan, Mohammad B. Alsavaf, Mohammed Alwabili, Jaskaran S. Gosal, Govind S. Bhuskute, Claudio Callejas, Kyle K. VanKoevering, Kyle C. Wu, Daniel M. Prevedello, Ricardo L. Carrau

**Affiliations:** ^1^ Department of Otorhinolaryngology–Head and Neck Surgery Tanta University Tanta Egypt; ^2^ Department of Otolaryngology–Head and Neck Surgery The Ohio State University Wexner Medical Center Columbus USA; ^3^ Department of Otolaryngology and Head–Neck Surgery Hospital Kuala Lumpur, Jalan Pahang 50586 Kuala Lumpur, Wilayah Persekutuan Kuala Lumpur Kuala Lumpur Malaysia; ^4^ Department of Neurological Surgery The Ohio State University Wexner Medical Center Columbus USA; ^5^ Department of Otorhinolaryngology–Head and Neck Surgery Prince Sultan Military Medical City Riyadh Saudi Arabia; ^6^ Department of Neurosurgery All India Institute of Medical Sciences (AIIMS) Jodhpur Rajasthan India; ^7^ Department of Otolaryngology All India Institute of Medical Sciences (AIIMS) Patna Bihar India

**Keywords:** clivus, craniovertebral junction, nasoseptal flap, skull base defect, skull base reconstruction

## Abstract

**Objective:**

Skull base defects can be challenging to reconstruct. The nasoseptal flap (NSF) remains the first‐line option for reconstruction. However, it can be inadequate to cover wide defects or compromised by tumor invasion or prior surgery requiring additional reconstructive options. The goal of the study is to describe a novel flap for clival and craniovertebral junction (CVJ) reconstruction.

**Study Design:**

Cadaveric study with an illustrative clinical case.

**Setting:**

Cadaver dissection laboratory and tertiary university hospital.

**Methods:**

Endoscopic endonasal dissection was performed in 15 cadavers. A modification of the inferior incision of the NSF was carried out. The inferior incision was performed at a more cranial location, sparing the mucosa of the posterior septum. Two lateral longitudinal incisions were carried out at the pterygo‐sphenoidal junction freeing the nasopharyngeal mucosa. A third incision was performed separating the rostral mucosa from the nasal floor. The resulting flap was rotated postero‐superiorly covering the clivus and CVJ.

**Results:**

An inferiorly based butterfly‐shaped nasopharyngo‐septal flap, consisting of nasopharyngeal and posterior septal mucosa and receiving blood supply from the bilateral ascending pharyngeal arteries, was formulated. The lower wings comprised nasopharyngeal mucosa while the upper wings comprised posterior septal mucosa. The mean surface area of the flap was 12.35 ± 0.21 cm^2^ covering the clivus and CVJ in all cadavers.

**Conclusion:**

The nasopharyngo‐septal flap is a novel vascularized flap that is well‐suited for reconstructing clival and CVJ defects where the NSF is insufficient and can also be used as a salvage flap in cases where the NSF is unobtainable.

Extended endoscopic endonasal approaches (EEA) have revolutionized skull base surgery by providing a less disruptive route to access and resect select ventral skull base lesions including those situated in the clival and craniovertebral junction (CVJ) regions. However, resulting skull base defects are challenging to reconstruct with the highest rates of reconstructive failures, because of their large size and lack of a sturdy repair matrix for adequate coverage of key neurovascular structures.^1^, ^2^ In addition, the inferior extension and vertical orientation of CVJ defects require increased reach to avoid vascular pedicle torsion. The nasoseptal flap (NSF) is a pedicled vascularized flap that has proven its reach and reliability; thus, earning its status as a “work‐horse” for skull base reconstruction.^3^ Recent modifications such as the unilateral and bilateral “rescue flap” approaches allow for the inferolateral displacement of the mucosa of the posterior septum and sphenoid face to facilitate maximum bony exposure without injury to the NSF pedicle.^4^, ^5^ Other septal mucosa‐preserving approaches to resect clival and CVJ lesions with good outcomes have also been reported.^6^, ^7^, ^8^ However, in case of wide skull base defects, the coverage provided by the NSF may be inadequate for the lower third of the clivus and CVJ following a panclivectomy. Furthermore, the NSF may be impractical if its pedicle or mucosal paddle has been compromised, or is unavailable in revision surgery. This cadaveric study describes a novel nasopharyngo‐septal butterfly flap (BFF) for the reconstruction of clival and CVJ defects if the NSF has inadequate lower reach. In addition, it can also be used as a salvage option.

## Materials and Methods

### Materials

Endoscopic endonasal dissection of 15 adult human cadaveric specimens was performed at the Anatomy Laboratory Toward Visuospatial Surgical Innovations in Otolaryngology and Neurosurgery (ALT‐VISION) at the Wexner Medical Center of The Ohio State University. The common carotid and vertebral arteries of all specimens were isolated, cannulated, and flushed with warm tap water to clear blood clots over 3 sessions. Subsequently, vessels were injected with red silicone for the arterial system. All specimens were kept in a 70% alcohol solution throughout the procedure. Rod‐lens endoscopes (4 mm diameter, 18 cm length) with 0° and 30° lenses (Karl Storz Endoscopy; Karl Storz) were paired with a high‐definition camera and video monitor. Video and digital images of the dissections were recorded using the AIDA recording system (Karl Storz Endoscopy; Karl Storz). Five screws were placed into every cadaveric cranium, and a high‐resolution multiplanar 1 mm thin‐cut computed tomography (CT) scan was performed for every cadaver before the dissection. Data was exported to a surgical navigation system (Stryker, iNtellect image guidance) to obtain stereotactic measurements of the anatomical field during dissection. Curved and straight instruments, along with a high‐speed drill (Medtronic) were used during the dissection. Dissections were performed according to rules and regulations for human tissue handling on de‐identified cadaveric specimens, and therefore the study was exempted from the institutional review board of The Ohio State University.

### Methods

#### Surgical Technique

##### NSF Harvest Modification

**Figure 1:**
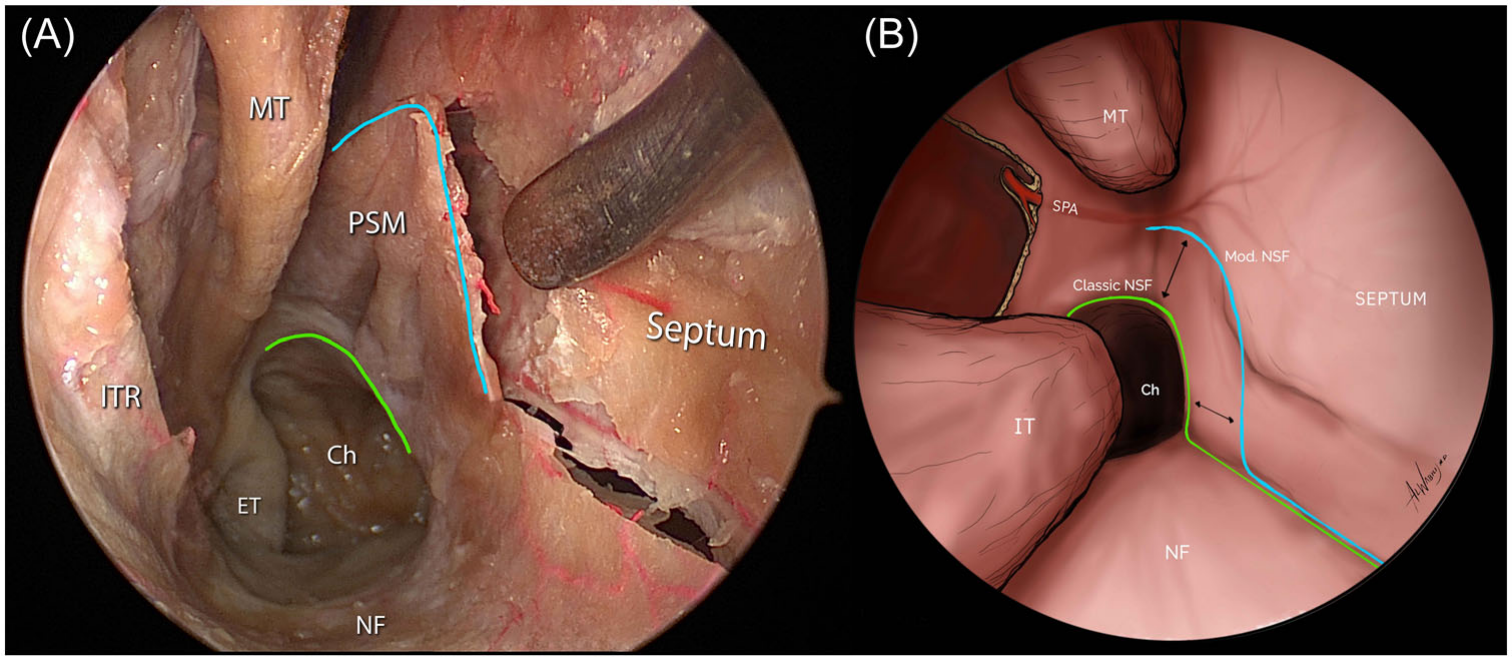
Illustrating NSF modification. (A) Demonstrates endoscopic endonasal view of cadaveric dissection of the nasoseptal flap with an elevated inferior incision sparing the posterior septal mucosa. (B) Diagram illustrating the classic inferior incision of the NSF passing across the upper choanal margin highlighted in green, and the modified incision line sparing the posterior septal mucosa highlighted in light blue. Ch, choana; ET, eustachian tube; IT, inferior turbinate; ITR, inferior turbinate remnants; MT, middle turbinate; NF, nasal floor; PSM, posterior septal mucosa; SPA, sphenopalatine artery.

The superior incision was made just underneath the sphenoid ostium that was identified before making the incisions. This was carried forward onto the nasal septum at a level approximately 1.5 cm below and parallel to the skull base (avoiding the olfactory epithelium). The incision was carried forward along the septum towards the mucocutaneous junction. The incision was then carried downwards vertically, terminating at a point where it connected with the planned inferior aspect of the flap. Following this, the inferior incision was made at a more superior level sparing the mucosa over the sphenoid rostrum instead of passing through the choanal margin. This was carried down onto the septum, sparing a 1.5 cm mucosal strip attached over the vomer. This ensured that the septal mucosal component of the BFF remained in continuity with the mucosa of the nasopharynx roof. The incision was then carried forward along a line just above and parallel to the maxillary crest, all the way forward to meet the vertical limb of the incision. Lastly, the NSF flap was elevated from anterior to posterior using a Cottle elevator along a subperichondrial and subperiosteal plane back to the anterior face of the sphenoid sinus (Figure 1).

##### Transclival Corridor and BFF Elevation Technique

The BFF was formed by 3 components of mucosa: (1) posterior septal, (2) sphenoid rostrum and nasopharyngeal roof, and (3) nasopharyngeal wall.

**Figure 2:**
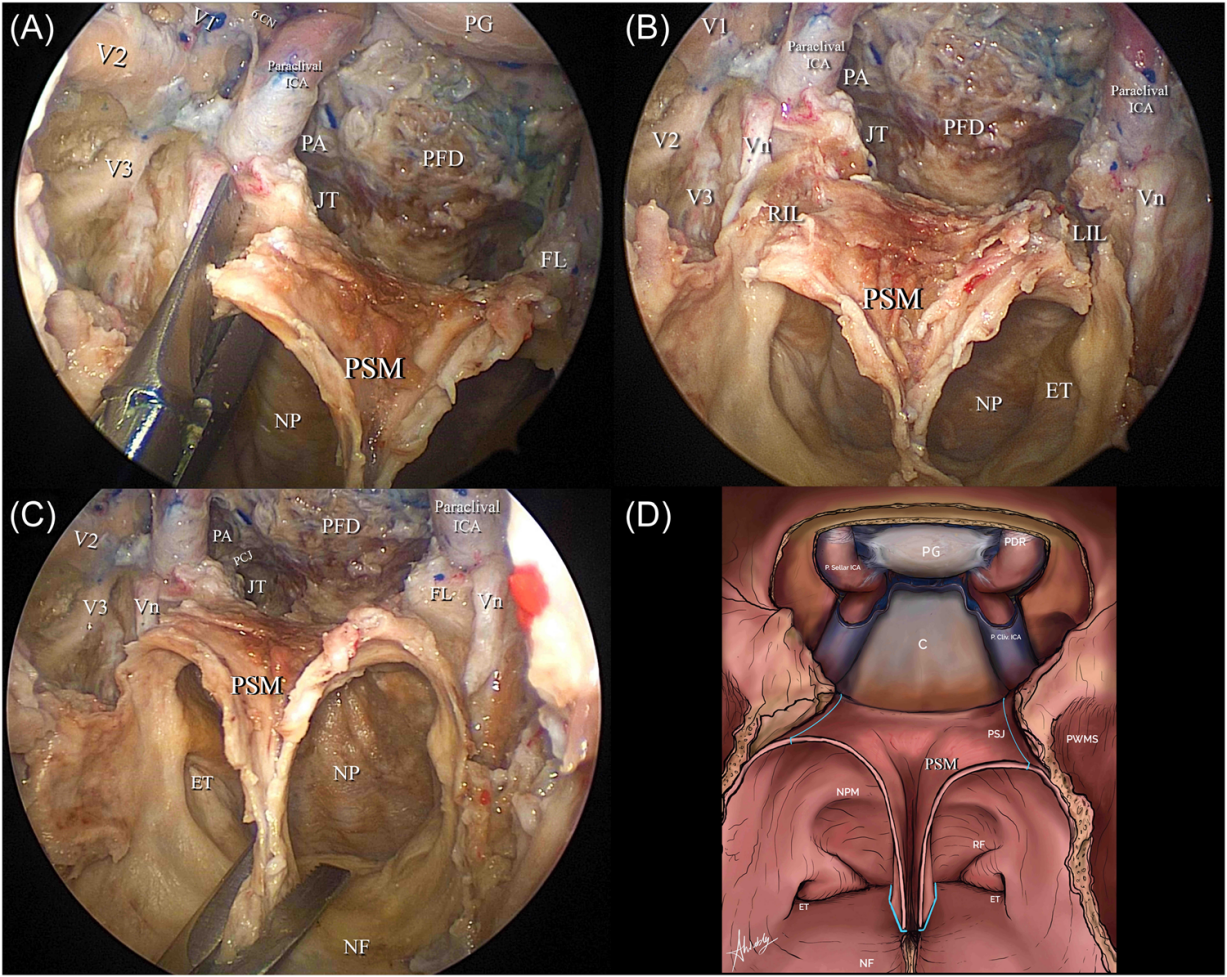
Butterfly flap Incisions. (A) Shows the right lateral vertical incision passing through the right pterygosphenoidal junction. (B) Shows the 2 lateral incisions freeing posterior septal and nasopharyngeal roof mucosa. (C) Shows the third inferior incision separating the posterior septal mucosa from the nasal floor mucosa. (D) An illustrative diagram showing the 3 incisions performed to free the flap‐highlighted in light blue. 6 CN, abducens nerve; C, clivus; ET, eustachian tube; FL, foramen lacerum; JT, jugular tubercle; LIL, left incisional line; NF, nasal floor; NP, nasopharynx; NPM, nasopharyngeal mucosa; PA, petrous apex; PDR, proximal dural ring; PFD, posterior fossa dura; PG, pituitary gland; PSJ, pterygosphenoidal junction; PSM, posterior septal mucusa; PWMS, posterior wall of maxillary sinus; RF, rosenmuller fossa; RIL, right incisional line; V1, ophthalmic division of trigeminal nerve; V2, maxillary division of trigeminal nerve; V3, mandibular division of trigeminal nerve; Vn, vidian nerve.

The posterior septectomy was completed, resecting the perpendicular plate of the ethmoid bone and vomer flush to the nasal cavity floor. Thus, the rostrum and sphenoidal floor were exposed with preservation of the bilateral mucosal strips. Extended sphenoidotomies with removal of the anterior wall of the sphenoid sinus were performed. The septal mucosal strips were then released anteriorly, followed by elevation of the “V” shaped mucoperiosteum overlying the sphenoid keel. The flap was released further, continuing to the roof of the nasopharynx. These maneuvers have completed the raising of the first 2 components of the flap. Finally, bilateral vertical incisions were made along the pterygosphenoid junction medial to the vidian canals extending posteriorly and downward along the petroclival synchondrosis. The dissection of the flap continued caudally, freeing it from the longi capiti muscles to reach the level of the nasal floor. This completed the elevation of the final component of the BFF. The sphenoid sinus floor was drilled followed by removal of the clivus to the CVJ, exposing the posterior fossa dura with skeletonization of paraclival ICAs bilaterally. Foramen lacerum, pterygosphenoidal junction, petroclival synchondrosis, odontoid process, C1 vertebra arch, eustachian tube orifices, and fossae of rosenmuller were exposed and identified. A third incision separating the posterior septal mucosa from the nasal floor mucosa was performed and the flap was rotated postero‐superiorly. The elevated flap could be displaced into the oropharynx with the bulk below the soft palate to keep it clear of the surgical field (Figure 2).

## Results

**Figure 3:**
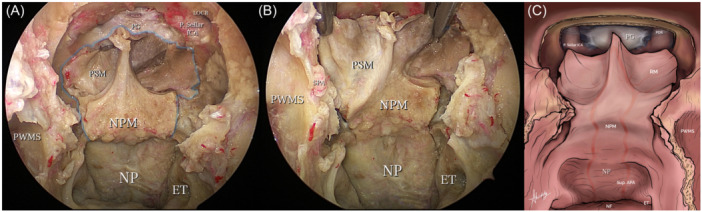
Extension and outlines of the butterfly flap. (A) Demonstrates flap boundaries highlighted in blue and consisting of nasopharyngeal and posterior septal mucosa. (B) Shows the flap held bilaterally straightening the mucosal folds and maximizing its area. (C) An illustrative diagram showing the flap extension. ET, eustachian tube; LOCR, lateral opticocarotid recess; NF, nasal floor; NP, nasopharynx; NPM, nasopharyngeal mucosa; PG, pituitary gland; PSM, posterior septal mucosa; PWMS, posterior wall of maxillary sinus; Sup. APA, superior branch of ascending pharyngeal artery.

**Figure 4:**
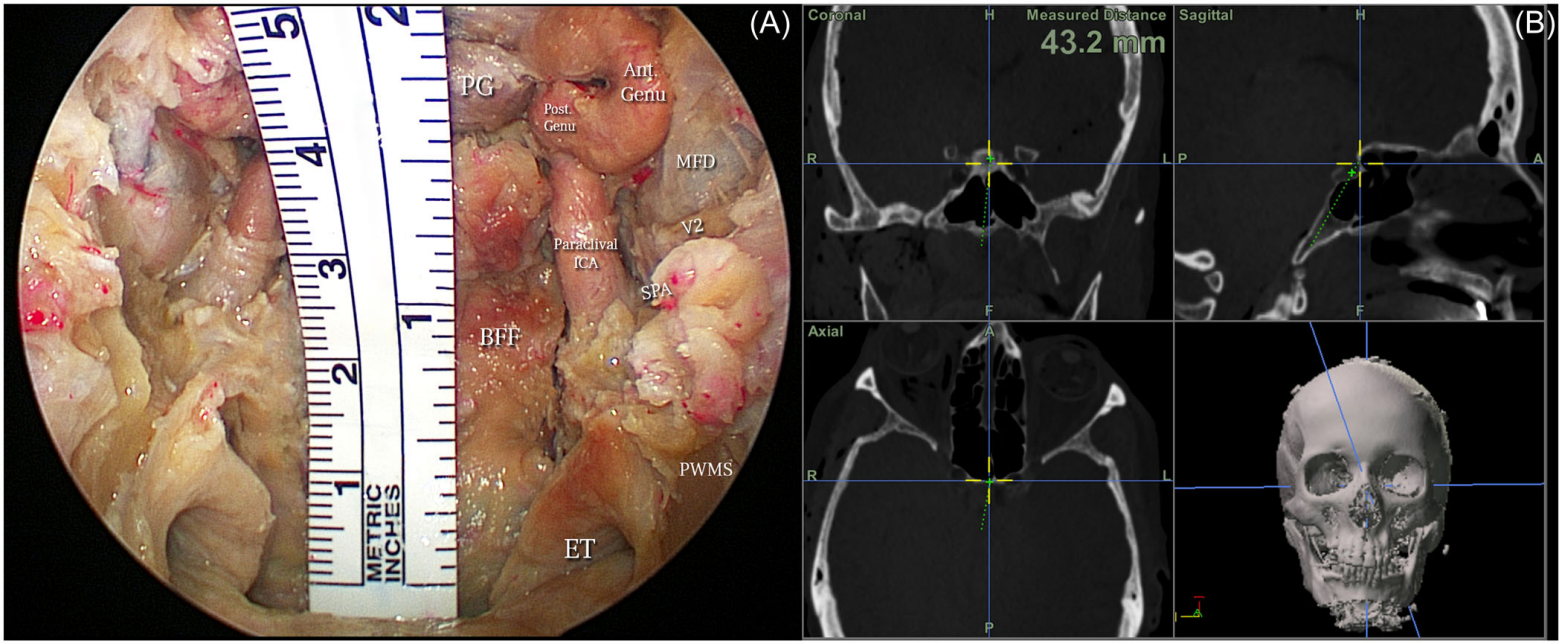
Illustrates the length and surface area covered by the flap. (A) Shows the length of the flap measured by a ruler ~4 cm covering the entire craniovertebral junction (CVJ) and clivus reaching the lower border of the pituitary gland. (B) Shows the length of the flap measured by the image‐guidance system to be 39.2 mm. Ant. Genu, anterior genu of the cavernous segment of the internal carotid artery; BFF, butterfly flap; ET, eustachian tube; MFD, middle fossa dura; PG, pituitary gland; Post. Genu, posterior genu of the cavernous segment of the internal carotid artery; PWMS, posterior wall of maxillary sinus; SPA, sphenopalatine artery; V2, maxillary division of trigeminal nerve.

The morphometric analysis of the BFF revealed that the mean length was 4.74 ± 0.96 cm and the width measurements showed a mean of 2.79 ± 0.59 cm. The surface area of the BF flap was measured, yielding a mean of 12.35 ± 0.21 cm². It achieved adequate coverage over the CVJ inferiorly, and additionally over the inferior and middle clival regions (Figures 3 and 4). The mean surface area of NSF coverage was measured as 20.89 ± 3.03 cm^2^, while the NSF combined with the BFF exhibited a significantly larger mean surface area of 33.24 ± 3.24 cm^2^. The difference between the 2 groups was statistically significant, with a *P *< .001 (Table 1).

**Table 1:** Morphometric Analysis of the Butterfly Flap

Butterfly flap measurements	
Length of the butterfly flap	4.74 ± 0.96 cm
Width of the butterfly flap	2.79 ± 0.59 cm
Surface area of the butterfly flap	12.35 ± 0.21 cm^2^

	Nasoseptal flap	Nasoseptal flap + Butterfly flab	*P* value
Surface area	20.89 ± 3.03 cm^2^	33.24 ± 3.24 cm^2^	<.001

The shows measurements of the length, width, and surface area of the butterfly flap when used alone and in combination with the nasoseptal flap.

Besides having a wider surface area of coverage, an extra advantage of having the 2 flaps was the ability to achieve multiple possible configurations and axes of rotation for better skull base reconstruction (Video 1).

## Illustrative Case

A 41‐year‐old male presented with a large right petroclival meningioma. Preoperative MRI showed a large dural‐based enhancing mass at the right central skull base measuring 3.9 × 5.1 cm. The lesion exerted a mass effect upon the right aspect of the brainstem, anterior aspect of the right cerebellar hemisphere, medial right temporal lobe, undersurface of the right cerebral hemisphere, and right optic radiations. It also enveloped the basilar artery by approximately 270° of its circumference. The patient underwent surgical debulking of the tumor to reduce brain stem compression via an endoscopic endonasal approach. The operation started with right NSF elevation and the flap was kept along the lateral wall of the right nasal cavity using silk sutures, which was followed by a left‐side reverse NSF to resurface the right‐side septal cartilage donor site. The nasal corridor was then prepared in the standard fashion to ensure wide exposure from the planum sphenoidale to the lower clivus and laterally from carotid to carotid.

The nasopharyngo‐septal BFF was then elevated. Incisions were made with monopolar cautery along the roof of the nasopharynx mucosa on each side and then these incisions were extended inferiorly. The flap was raised with monopolar cautery to encompass the nasopharyngeal roof and the posterior nasopharyngeal wall mucoperiosteum. Following flap release, a suture was placed to ensure it remained in the oropharynx during the surgery. A mass was identified within the right sphenoid sinus and was confirmed by a fresh frozen section to be consistent with a meningioma.

**Figure 5:**
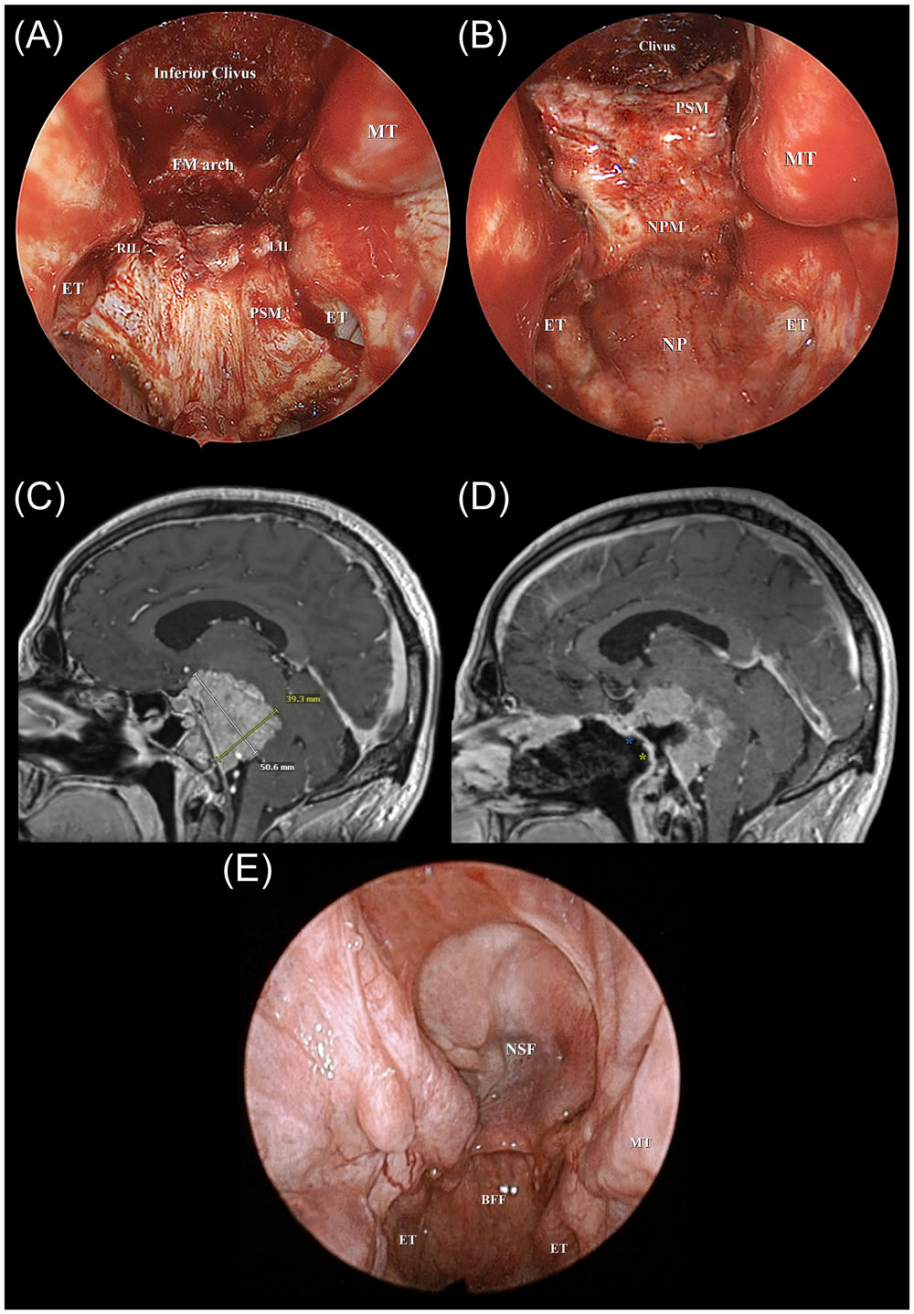
Shows a case of petroclival meningioma. (A) An endoscopic endonasal view showing BFF incisions. (B) An endoscopic endonasal view showing BFF rotated postero‐superiorly towards the clivus. (C) Preoperative T1 weighted MRI with contrast showing the petroclival meningioma measuring about 3.9 × 5.1 cm and compressing the brain stem. (D) Postoperative T1 weighted MRI with contrast showing central tumor debulking and reconstruction with the nasoseptal flap superiorly (blue asterisk) and the butterfly flap inferiorly (yellow asterisk) overlapping at mid‐clivus, both flaps show contrast enhancement. (E) An endoscopic endonasal view showing healing of both nasoseptal and butterfly flaps 1 month postoperatively. BFF, butterfly flap; ET, eustachian tube; FM arch, foramen magnum arch; LIL, left incisional line; MT, middle turbinate; NP, nasopharynx; NPM, nasopharyngeal mucosa; NSF, nasoseptal flap; PSM, posterior septal mucosa; RIL, right incisional line.

A transclival approach to the posterior and middle fossa was then commenced followed by central tumor debulking. A Grade 3 intraoperative CSF leak was noted after dural opening. Multilayer reconstruction was concluded by repositioning the BFF and the NSF using an onlay technique on the exposed bone surrounding the defect. Both flaps abutted at the middle clivus region. The postoperative MRI showed a reduction in the central volume of the tumor and improvement in the midbrain and pontine deformity compared to preoperative imaging. At 6‐month postoperative follow‐up, there was no evidence of postoperative CSF leak, mucocele, or meningitis with good mucosalization of donor and recipient sites (Figure 5).

## Discussion

Despite the extirpative options afforded by EEAs, clival reconstruction after surgery remains a challenge. High pressure and flow of CSF in the prepontine cistern, and frequent need for postoperative use of radiation therapy often requires a complex and sturdy repair. CSF leak rates following transclival approaches have been reported from 19% to 41% compared to a 4.2% reconstructive failure rate in patients who underwent CVJ surgery.^9^, ^10^ Pontine encephaloceles can also occur in cases with extensive resection of the clivus.^11^, ^12^

Existing reconstruction options utilizing an intranasal vascularized flap are limited if the NSF is inadequate. The extended inferior turbinate flap (ITF) has been used by surgeons in such cases; however, mobilization of the pedicle and placement of the flap to ensure adequate closure is technically demanding.^13^ The lateral nasal wall flap (LNWF) has also been shown to be suitable for posterior fossa reconstruction given the pedicle location and orientation, however, the specific success rate was unable to be discerned due to a lack of subgroup analysis.^14^ Another options are combination flaps, however, there is a possibility of suboptimal reconstruction due to interruption in coverage area between the components of the 2 separate flaps.^15^ Other vascularized flaps of extranasal origin, such as the temporalis fascia flap or the pericranial flap may be necessary.^16^ These flaps are more complex to harvest, position and require a longer recovery time. Alternatively, an inferiorly based flap, the rhinopharyngeal flap (RPF) has been in use to address lower clivus and CVJ defects.^17^ While it has been shown to provide adequate vascularized coverage, it did not have a demonstrable effect on CSF leak rates.^18^

The BFF derives its vascular supply from bilateral ascending pharyngeal arteries of the external carotid artery (ECA) system. The flap is designed so that despite the situation of the pedicle in the inferior portion, bilateral supply maintains an overlap in vascularization; ensuring that the superior portion of the flap remains viable with less possibility of flap contracture and more chances of flap viability. It also may receive anastomotic blood supply from the recurrent artery of the foramen lacerum, meningohypophyseal trunk of the cavernous segment of the internal carotid artery, or descending branches of the lesser palatine artery.^19^, ^20^ As such, mucosa overlying the keel and posterior septum can be harvested as part of the flap, increasing its reach. Such positioning of the pedicle also allows for easy placement of the paddle to cover posterior fossa defects. As it is harvested from bone and cartilage that is subsequently removed, it should not be associated with crusting due to exposed surfaces. Most of the flap incision lines are also accomplished during the exposure of the lesions addressed. Furthermore, the posterior location leads to decreased donor site morbidity from a rhinological standpoint, as nasal air conditioning and olfaction are not affected. Moreover, the soft palate muscles are kept intact to avoid any risk of velopharyngeal insufficiency. The technique of raising and positioning the BFF is much less complex compared to an ITF or LNWF.^13^, ^21^ However, the posterior dissection of the flap requires diathermy and should follow the bone of the clivus, incorporating pharyngobasilar fascia and longus capitis muscle into the flap. It is also important to utilize Doppler ultrasound or image guidance to map the position of the parapharyngeal segment of the internal carotid arteries bilaterally and confirm its position with respect to the margins of the flap before making the mucosal cuts. The BFF has a larger reconstructive surface in comparison with other flaps based on a separate pedicle from the sphenopalatine artery and its branches (Table 2). We posit that in cases of tumors affecting clivus, petrous, foramen magnum, and CVJ regions with lateral, superior, or inferior extensions where there can be very large defects, the BFF also provides an excellent option for reconstruction when used in combination with the NSF as the pedicle is distinct. In our case, the large size of petroclival meningioma required reconstruction of a defect extending from the planum sphenoidale superiorly to the lower third of the clivus inferiorly. The lateral location of the NSF pedicle allowed reconstruction in an upward or downward fashion from the origin of its vascular supply which was roughly situated in the middle of the region that required reconstruction, thus the NSF was unable to be used as a singular option. The NSF–BFF amalgamation proved to be a good combination with the 2 flaps joining at the middle clivus. Moreover, revision surgeries for lesions such as nasopharyngeal carcinomas, clival chordomas, petroclival chondrosarcomas, and meningiomas would benefit from the availability of this option.

**Table 2:** Local Vascularized Flaps for Clival Reconstruction

Flap	Reconstruction area	Pedicle
ITF (posterior pedicled)^21^	Upper clivus	Posterior lateral nasal artery and descending palatine artery
Extended ITF^13^	Upper and middle clivus	Posterior lateral nasal artery and descending palatine artery
Basipharyngeal flap^22^	Lower clivus	Bilateral ascending pharyngeal artery
Upper tongue flap^23^	Lower and middle clivus	Bilateral ascending pharyngeal artery
Rhinopharyngeal flap^17^	Lower and middle clivus	Bilateral ascending pharyngeal artery
Butterfly flap (current study)	CVJ, lower to upper clivus, and sella^a^	Bilateral ascending pharyngeal artery

The table shows different reconstruction areas and pedicles of local vascularized flaps used for clival reconstruction.

^a^ If nasal floor mucosa is included.

**Figure 6:**
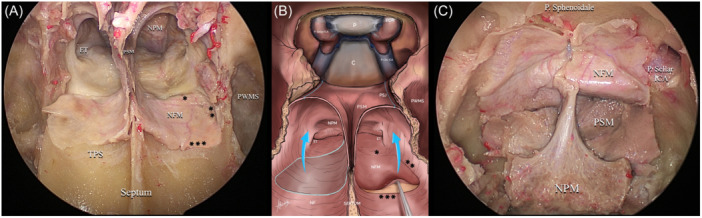
Shows the addition of nasal floor mucosa to the flap. (A) Demonstrates 3 bilateral incisions added to separate the floor mucosa; * first horizontal incision is made at the junction between soft and hard palate, ** a second vertical incision is made separating the nasal floor from lateral wall mucosa, and *** a third horizontal incision is made at the bony suture between the palatine process of maxilla and horizontal plate of palatine bone (transverse palatine suture). (B) An illustrative diagram showing the 3 incisions used to harvest nasal floor mucosa and the direction of flap rotation. (C) Shows flap parts and extension covering the whole clivus and sella up to planum sphenoidale. C, clivus; ET, eustachian tube; NF, nasal floor; NFM, nasal floor mucosa; NPM, nasopharyngeal mucosa; P, pituitary gland; PDR, proximal dural ring; PSJ, pterygosphenoidal junction; PSM, posterior septal mucosa; PWMS, posterior wall of maxillary sinus; P. sphenoidale, planum sphenoidale; TPS, transverse palatine suture.

The mucosa from the floor of the nose can also be incorporated, added as an extension of the posterior septal mucosa on both sides. This would result in an addition of an average surface area of 4.112 ± 0.76 cm^2^ of mucosa and extends the coverage to the sellar region superiorly, however, that portion would have to be placed over dura or periosteum to acquire blood supply (Figure 6).

## Limitation

The BFF has limited usage in revision cases where the traditional NSF has been harvested without preservation of the posterior septal mucosa. The BFF demonstrated utility in reconstructing a large clival defect in conjunction with an NSF in 1 patient. However, more cases are needed to prove and cement its function as a solitary repair option for this region as well.

## Conclusion

The nasopharyngo‐septal BFF is a novel vascularized flap that provides a large surface area of viable tissue for the reconstruction of skull base defects in the clival and CVJ regions. Its potential role in salvage surgery and effectiveness in preventing CSF leakage remains to be confirmed in more clinical patients.

**Video 1:** Transcription

## Author Contributions

**Moataz D. Abouammo**, conceptualization, cadaveric dissections, manuscript writing; **Maithrea S. Narayanan**, cadaveric dissections, manuscript writing; **Mohammad B. Alsavaf**, cadaveric dissections and data analysis; **Mohammed Alwabili**, data analysis and illustrations; **Jaskaran S. Gosal**, cadaveric dissections, data collection; **Govind S. Bhuskute**, cadaveric dissections, data collection; **Claudio Callejas**, review and revision; **Kyle K. VanKoevering**, review and revision; **Kyle C. Wu**, review and revision; **Daniel M. Prevedello**, supervision, resources, review and revision; **Ricardo L. Carrau**, supervision, resources, review and revision.

## Disclosures

### Competing interests

None.

### Funding source

None.
